# Surgery for retrohepatic caval thrombus in patients with advanced renal cell carcinoma: a case series

**DOI:** 10.1186/s12957-015-0765-5

**Published:** 2016-01-15

**Authors:** Małgorzata Polańska-Płachta, Robert Proczka, Magdalena Dudek, Małgorzata Ostrowska, Jerzy A. Polański

**Affiliations:** 1 2nd Department of General, Vascular and Oncologic Surgery, 2nd Faculty of Medicine with English Division and Physiotherapy Division, Czerniakowski Hospital, Medical University of Warsaw, Stepinska Str. 19/25, 00-739 Warsaw, Poland; 2 Department of Urology, 1st Faculty of Medicine, Medical University of Warsaw, Lindleya Str. 4, 02-005 Warsaw, Poland

**Keywords:** Carcinoma, renal cell/surgery, Vena cava, inferior, Venous thrombosis/surgery

## Abstract

**Background:**

Thrombotic involvement of the inferior vena cava (IVC) occurs in about 10 % of all patients with renal cell carcinoma (RCC). It is treated with radical resection of tumor and thrombus. We present the results of a recent case series of 20 patients with retrohepatic IVC thrombus.

**Methods:**

Our cohort of 20 patients included 16 primary resections (radical nephrectomy and thrombectomy with and without vascular graft), three recurrences primarily operated on elsewhere (thrombectomy and vascular graft), and one recurrence due to a new liver metastasis.

**Results:**

All surviving patients were discharged with a patent IVC. The overall mortality rate was 10 %, and the overall complication rate was 35 %. Both are in keeping with results presented worldwide.

**Conclusions:**

Our series provides a corroborating extension to the existing dataset on RCC-related IVC thrombus removal. It confirms that the radical surgical approach can be performed safely and successfully with respect to venous patency.

## Background

**Fig. 1:**
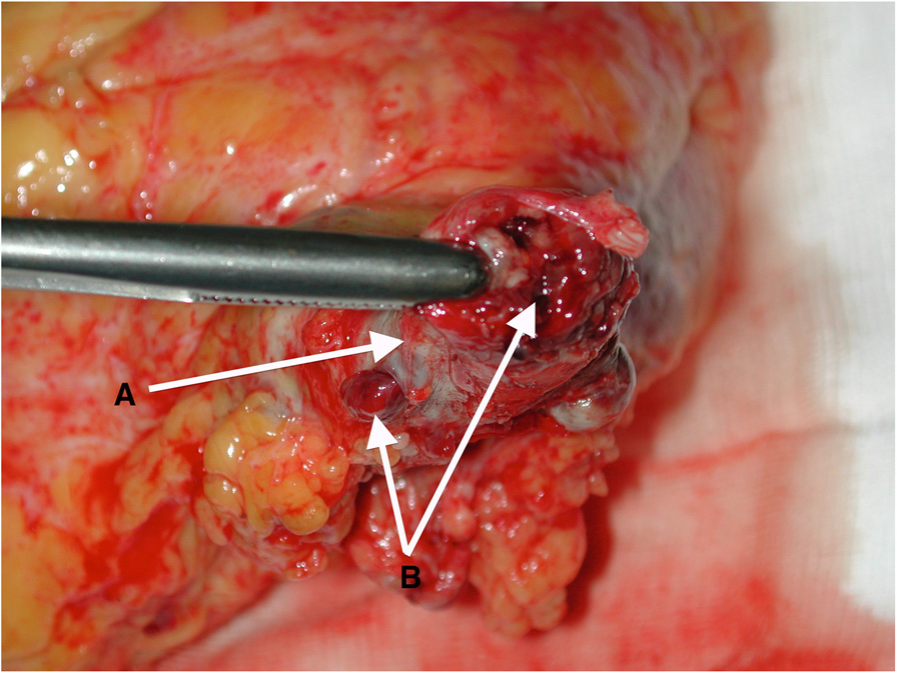
Left renal vein totally occluded by neoplastic thrombus. **a** Wall of the renal vein. **b** Neoplastic thrombus

Venous involvement, manifesting as tumor thrombus in the renal vein on the affected side and potentially extending into the inferior vena cava (IVC) or even the right atrium is a well-known complication of renal cell carcinoma (RCC) (Fig. 1). That last category requires cardiac surgery input due to the requirement for cardiopulmonary bypass. The overall incidence is estimated to be up to 10 % of all renal cancer patients [1] with IVC involvement more common on the right because the renal vein is shorter on that side [2] (Fig. 1).

The importance of neoplastic venous thrombosis is both symptomatic and prognostic. In addition to the usual symptoms of RCC (flank pain, hematuria, and symptoms from metastases), patients also suffer the unpleasant sequelae of IVC obstruction—sometimes referred to as IVC syndrome—including marked edema of the IVC-drained territories and hemodynamic changes due to reduced preload.

While the exact prognostic value of venous involvement is still debated, a few suggestions regarding its importance have been made. Five-year survival rates for cancer involving the venous system exceeds 50 %—with reported ranges varying widely from 25 to 70 % [3]. The characteristics of the underlying tumor, rather than the presence or level of thrombus, seem to determine prognosis [4]. More importantly, the long-term survival of patients in whom complete resection is achieved appears to be markedly better compared to cases with incomplete resection [5]. Recurrence of the thrombosis thus seems to originate from residual thrombus left after incomplete resection.

**Fig. 2:**
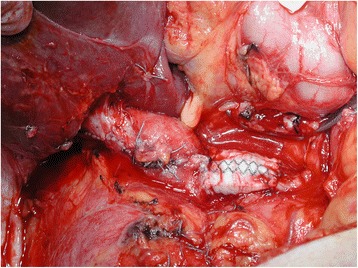
Retrohepatic inferior vena cava (IVC) closed with aid of a Dacron® graft

From a surgical perspective, involvement of the retrohepatic portion of the IVC presents unique technical challenges. The fragility of the thrombi requires excision under direct vision, necessitating extensive exposure of the retrohepatic IVC by mobilizing the right liver lobe, dividing its ligaments, and retracting it to the left (Fig. 2).

The extension of the approach of radical tumor resection to cases with venous involvement is generally considered to be justified based on available data. Indeed, it is the treatment of choice as practiced by many centers worldwide. Most importantly, the treatment is best carried out in centralized, tertiary referral centers with the involvement of a multidisciplinary team and meticulously tailored perioperative management [6].

We present a case series of patients who were diagnosed with RCC-related IVC thrombosis in the retrohepatic portion of the vessel. Our report will complement the currently available outcome data with respect to the surgical approach.

## Methods

### Patients

From 2007 to 2014, a cohort of 20 patients with RCC-related venous thrombus in the retrohepatic portion of the inferior vena cava (IVC) underwent surgical thrombectomy. Diagnosis was established through computed tomography and ultrasound. Sixteen surgeries were primary interventions with concomitant ipsilateral radical nephrectomy. Four cases were reoperations due to recurrent thrombus, three of which had previously been operated on in other centers. Time from the primary operation to presentation at our department ranged from 3 months to 5 years. The remaining patient who had been treated in our department 5 years earlier, with nephrectomy and thrombus removal from the retrohepatic portion of the IVC, developed thrombus of the retrohepatic IVC due to a left liver lobe metastasis.

Of patients, 30 % (*n* = 6) were female and 70 % (*n* = 14) were male. Age at time of surgery ranged from 35 to 73 years, with an average of 56 years. The original presenting symptoms were hematuria in 75 % of patients (*n* = 15), weight loss in 45 % (*n* = 9), and flank pain in 30 % (*n* = 6); 20 % (*n* = 4) had a past history of pulmonary embolism (PE), and 30 % (*n* = 6) had suffered deep venous thrombosis (DVT) in the lower limbs and IVC below the level of the renal veins; 35 % had chronic cardiac conditions, 20 % were diabetic, and 30 % were hypertensive. Among the 16 patients who underwent radical nephrectomy, 2 patients had a left-sided tumor (12.5 %) and 14 (87.5 %) had a right-sided tumor. Average tumor size was 13.4 cm, ranging from 6.7 to 16.9 cm. All thrombi extended into the retrohepatic portion of the IVC. Patient data are detailed in Table 1.

**Table 1:** Demographic data and disease factors in patients with advanced renal cell carcinoma and retrohepatic caval thrombus

Variable	Number of patients	Percent (%)
Sex		
M	14	70
F	6	30
Age in years		
Mean	56	
Range	35–73	
Symptoms		
Hematuria	15	
Weight loss	9	
Flank pain	6	
Prior pulmonary embolism (PE)	4	
Deep venous thrombosis (DVT) below renal veins	6	
Localization of tumor		
Left kidney	2	
Right kidney	14	
Left lobe of liver	1	
Size of tumor within kidney		
Mean	13.4	
Range	6.7–16.9	
Thrombus level within inferior vena cava (IVC)	Retrohepatic	100
Distant metastases	6	
Lymph node involvement	17	
IVC wall infiltration	6	
Accompany diseases		
Chronic heart disease	7	
Diabetes	4	
Hypertension	6	

### Procedure

**Fig. 3:**
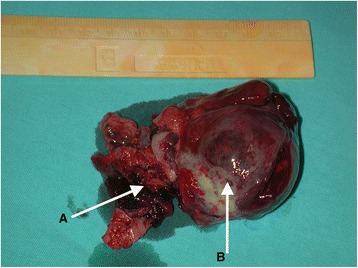
Neoplastic thrombus removed from IVC. **a** Neoplastic thrombus. **b** Kidney with neoplasm

Open operative access was obtained through an inverted T incision in the upper abdomen. The infrahepatic portion of the inferior vena cava (IVC) was exposed, and both renal veins were mobilized. The right liver lobe was mobilized in 19 cases and the left liver lobe in 1 case to provide access to the retrohepatic portion of the IVC. The vena cava dissection was carried out cephalad up to the level of the diaphragm. Intraoperative ultrasound was used to localize the thrombus within the lumen. The IVC was clamped inferior to the termination of the right renal vein. The renal vein on the unaffected side was also clamped. The hepatoduodenal ligament was clamped next, and then, the vena cava was clamped suprahepatically. Finally, the renal vein on the affected side was clamped. Cavotomy was performed via a longitudinal incision extending between the anterior surface of the affected renal vein at its point of drainage into the vena cava and extended to the level of the head of the thrombus as visualized by intraoperative ultrasound. The thrombus was excised under direct vision (Fig. 3), and the IVC wall was inspected for attachment and infiltration. In case of a positive finding, an IVC wall biopsy was taken for intraoperative pathological examination. The posterior wall of the affected renal vein was excised and the venocavotomy closed with continuous vascular suture. If the excised circumference of the vessel was more than half of the original circumference, the defect in the vessel wall was fixed using a polytetrafluoroethylene (PTFE) patch (*n* = 4 patients). In cases where the pathology report showed tumor infiltration of the IVC wall, the whole segment affected was resected and replaced with a 19-mm reinforced PTFE or Dacron® interposition graft (*n* = 2) (Fig. 3). After the blood flowed through the IVC and the liver was restored, a radical nephrectomy was performed on the affected kidney and renal vein. The specimen obtained was sent for histopathological examination. The retroperitoneum was assessed for hemostasis, and a drain was left in place.

All of the patients in the recurrence group underwent IVC resection with concomitant PTFE grafting. In order to achieve this, the retrohepatic segment of the IVC was exposed with ligation and division of all short liver veins up to their confluence with the right hepatic vein.

After clamping the IVC below the level of the right renal vein, the left renal vein, and the IVC immediately below its junction with the right hepatic vein, the affected segment of the IVC was removed along with the confluence with the renal vein on the affected side by diagonal incision and replaced with a PTFE patch or graft. The patient who presented with late metastasis to the left liver lobe also underwent a left hemihepatectomy as part of the procedure. All patients received 1 mg of fractionated heparin per kilogram of body weight daily during the postoperative period and at the time of discharge.

## Results

The overall mortality rate was 10 %. Of the two deaths that occurred, one patient undergoing a primary procedure died 5 days after surgery due to cardiopulmonary insufficiency. This patient was preoperatively diagnosed with chronic coronary disease and had a history of myocardial infarction 3 years prior to the operation. The other death was due to a brain stem stroke 2 days after surgery in a patient undergoing a reoperation. IVC patency at discharge was achieved in all surviving patients. One patient required reoperation to evacuate a postoperative hematoma. Two patients suffered small pulmonary emboli. Two patients suffered infection of the operative wound, which increased their hospital stay by 3 days. The average blood loss was 768 ml and ranged from 300 to 2380 ml. Three patients, whose blood loss exceeded 1000 ml and who had a history of chronic cardiac conditions, received blood transfusions. In the recurrent group (excluding the liver metastasis patient), 67 % (two of three patients) had a thrombus attached to the renal venous stump.

Of the intraoperative pathology reports, 4 %indicated tumor invasion into the IVC wall. These patients underwent extensive excision and subsequent replacement of the IVC wall with artificial material as described earlier. A more detailed breakdown is presented in Tables 2 and 3 for the reoperation group.

**Table 2:** Postoperative data of patients with advanced renal cell carcinoma and retrohepatic caval thrombus

	Number of patients	Percent (%)
Procedure for retrohepatic inferior vena cava (IVC) associating nephrectomy		
Open thrombectomy	14	70
Patch graft	4	20
Interposition	2	10
Blood loss		
Mean	768	
Range	300–2380	
Perioperative complications		
Wound infection	2	
Postoperative internal bleeding	1	
VCI thrombosis	0	
Pulmonary embolization	2	
Death	2	10
Acute IVC patency	18 (2 deaths, patients excluded)	100

**Table 3:** Reoperation group: recurrence of retrohepatic inferior vena cava (IVC) thrombus

Patient	Months since nephrectomy and first thrombectomy	Staging at first surgery	Staging at second surgery	Procedure	Hospital mortality
GJ	6	pT3N1M0	NxM0	IVC thrombectomy and PTFE graft	Day 2
KT	9	pT4N1M0	NxMx	IVC thrombectomy and PTFE patch	
MP	3	pT3NxMx	NxMx	IVC thrombectomy and PTFE patch	
TJ	61	pT4NxM0	T3NxM1	Left hemihepatectomy, IVC thrombectomy and PTFE patch	

*PTFE* polytetrafluoroethylene

## Discussion

Although IVC thrombus resection is a major operation, it can be performed relatively safely, considering the gravity of the underlying disease. Our mortality figure of 10 % corresponds well to the globally reported rates ranging from 2.7 to 13 % [3]. Our overall complication rate (including fatalities) of 35 % is also within the globally reported range. For instance, the series with the lowest mortality rate to date quotes a 30 % complication rate [5]. The overall results are also consistent with outcomes achieved in our department when treating neoplastic thrombus affecting other portions of the IVC [7].

All surviving patients were discharged with a patent IVC, confirming the technical success of the procedure. This is particularly reassuring, as the low pressure venous system is especially sensitive to any changes within its wall, necessitating close attention to treatment during the postoperative period as well as after discharge. Given our results, fractionated heparin injections seem effective in maintaining patency. It also seems that closure of the IVC defect by continuous suture, which reduces the final vessel diameter by less than 50 %, does not affect outcome in terms of postoperative patency. Similarly, our series confirms the excellent suitability of PTFE and Dacron® patching and interposition grafting for the reconstruction of the resected IVC [8].

The finding of thrombus on the renal vein stump in the recurrent group points to that recurrent thrombus coming from incomplete original resection. This led us to adopt the practice of more extensive renal vein resection during radical nephrectomy, with the aim of also including the IVC/renal vein junction. Furthermore, the case of late RCC metastasis to the left liver lobe and recurrence of neoplastic thrombus within the retrohepatic IVC attached to the tumor seems to support the propensity of RCC to venous invasion. Since the invasion in this case must have originated from the hepatic metastasis, it is the nature of the tumor rather than its location that determines its vascular-invasive properties.

## Conclusions

We demonstrated the safety and good technical outcome of RCC-related retrohepatic IVC thrombus resection in a large cohort of patients in a variety of complex surgical scenarios.

## Abbreviations

DVT: deep venous thrombosis
IVC: inferior vena cava
PE: pulmonary embolism
PTFE: polytetrafluoroethylene
RCC: renal cell carcinoma
